# Identification of a *Pseudomonas aeruginosa* PAO1 DNA Methyltransferase, Its Targets, and Physiological Roles

**DOI:** 10.1128/mBio.02312-16

**Published:** 2017-02-21

**Authors:** Sebastian Doberenz, Denitsa Eckweiler, Olga Reichert, Vanessa Jensen, Boyke Bunk, Cathrin Spröer, Adrian Kordes, Emanuela Frangipani, Khai Luong, Jonas Korlach, Stephan Heeb, Jörg Overmann, Volkhard Kaever, Susanne Häussler

**Affiliations:** a Institute for Molecular Bacteriology, Twincore GmbH, Center for Clinical and Experimental Infection Research, Hannover, Germany; b Department of Molecular Bacteriology, Helmholtz Centre for Infection Research, Braunschweig, Germany; c Leibniz Institute DSMZ-German Collection of Microorganisms and Cell Cultures, Braunschweig, Germany; d School of Life Sciences, Centre for Biomolecular Sciences, University of Nottingham, Nottingham, United Kingdom; e Pacific Biosciences, Menlo Park, California, USA; f Research Core Unit Metabolomics, Hannover Medical School, Hannover, Germany; Institut Pasteur

## Abstract

DNA methylation is widespread among prokaryotes, and most DNA methylation reactions are catalyzed by adenine DNA methyltransferases, which are part of restriction-modification (R-M) systems. R-M systems are known for their role in the defense against foreign DNA; however, DNA methyltransferases also play functional roles in gene regulation. In this study, we used single-molecule real-time (SMRT) sequencing to uncover the genome-wide DNA methylation pattern in the opportunistic pathogen *Pseudomonas aeruginosa* PAO1. We identified a conserved sequence motif targeted by an adenine methyltransferase of a type I R-M system and quantified the presence of N^6^-methyladenine using liquid chromatography-tandem mass spectrometry (LC-MS/MS). Changes in the PAO1 methylation status were dependent on growth conditions and affected *P. aeruginosa* pathogenicity in a *Galleria mellonella* infection model. Furthermore, we found that methylated motifs in promoter regions led to shifts in sense and antisense gene expression, emphasizing the role of enzymatic DNA methylation as an epigenetic control of phenotypic traits in *P. aeruginosa*. Since the DNA methylation enzymes are not encoded in the core genome, our findings illustrate how the acquisition of accessory genes can shape the global *P. aeruginosa* transcriptome and thus may facilitate adaptation to new and challenging habitats.

## IMPORTANCE

With the introduction of advanced technologies, epigenetic regulation by DNA methyltransferases in bacteria has become a subject of intense studies. Here we identified an adenosine DNA methyltransferase in the opportunistic pathogen *Pseudomonas aeruginosa* PAO1, which is responsible for DNA methylation of a conserved sequence motif. The methylation level of all target sequences throughout the PAO1 genome was approximated to be in the range of 65 to 85% and was dependent on growth conditions. Inactivation of the methyltransferase revealed an attenuated-virulence phenotype in the *Galleria mellonella* infection model. Furthermore, differential expression of more than 90 genes was detected, including the small regulatory RNA *prrF1*, which contributes to a global iron-sparing response via the repression of a set of gene targets. Our finding of a methylation-dependent repression of the antisense transcript of the *prrF1* small regulatory RNA significantly expands our understanding of the regulatory mechanisms underlying active DNA methylation in bacteria.

## INTRODUCTION

Investigations of DNA methylation have mainly addressed cytosine methylation by 5-methylcytosine methyltransferases (MTases) in eukaryotes, and much less work has been performed on the role of adenine methylation in bacteria (1). Likewise there are many studies demonstrating the role of DNA methylation in epigenetic control for eukaryotes. However, recently the regulatory role of bacterial DNA MTases has gained increasing attention. DNA MTases methylate either cytosine or adenine, thereby forming C5-methylcytosine, N^4^-methylcytosine, or N^6^-methyladenine, respectively (2, 3). Most DNA methylation reactions in prokaryotes are catalyzed by DNA MTases, which are part of restriction-modification (R-M) systems (2, 4). The type I R-M system is composed of three major subunits, which consist of a methyltransferase (MTase) for DNA modification (HsdM), a DNA-binding protein for specificity (HsdS), and a restriction endonuclease (HsdR) domain (5). The specificity subunit recognizes a specific DNA motif for methylation (3, 6) and has been shown to function together with a dimer of the MTase (7); there are also solitary DNA MTases that do not have a restriction enzyme counterpart. Examples of the latter are the N^6^-adenine MTase Dam, CcrM (8) and the C-5 cytosine methylase Dcm (9, 10), and VchM (9, 11). While R-M systems are known for their role in the defense against foreign DNA (10), DNA methylation enzymes also seem to play functional roles in the timing of DNA replication, chromosome partitioning, DNA repair, control of transposition, and conjugal transfer of plasmids and gene regulation (12–20, 61, 62). Methylation of the amino group of adenine has structural effects that can influence DNA-protein interactions, especially for proteins that recognize their cognate DNA binding sites by both primary sequence and structure (21, 22). Thus, adenine methylation is used as an epigenetic signal to modulate given DNA-protein interactions that might include the interaction of transcription factors with their target DNA (23).

*Pseudomonas aeruginosa* is a Gram-negative environmental bacterium that can be found in a large variety of habitats. *P. aeruginosa* is also an opportunistic pathogen that causes severe acute and devastating chronic infections of major clinical importance (24–27). Its extraordinary adaptability and flexible expression of a large gene repertoire have been the subject of intense study (28, 29). Recent advances in various “omics” technologies enable quantitative monitoring of bacterial genomes and their derivatives, such as RNA, protein, and metabolites, in a high-throughput manner and thus allow for the determination of variations on a genomic scale (30). However, DNA methylation patterns have not been considered within the developing-systems-level view of *P. aeruginosa* so far. Of note, although genomic analysis revealed multiple putative MTases within individual *P. aeruginosa* genomes, those genes form part of the accessory genome (31). This suggests that although methylation is commonly observed in the species *P. aeruginosa*, the genome-wide methylation patterns are strain specific and depend on the acquisition of specific MTases and their respective specificity domain subunit.

Sodium bisulfite treatment of DNA has fostered research on epigenetic control of DNA methylation in eukaryotes as it greatly facilitated detection of N^4^-methylcytosine on a genome-wide scale (32). The recently established single-molecule real-time (SMRT) sequencing technique offers the possibility to identify the complete set of methylated sequence motifs, including the widespread bacterial N^6^-methyladenine (m6A) within microbial genomes (33–35). Furthermore, information on the methylation state can be obtained (35). Thus, SMRT technology represents a powerful tool for characterization of the role of DNA methylation in a wide variety of bacteria and has paved the way for studies on the potential regulatory roles of adenine methylation (33, 36).

In this study, we used SMRT sequencing to provide the first insights into DNA methylation in the *P. aeruginosa* type strain PAO1. We identified methylated sites throughout the genome, as well as the sequence motif targeted by a predicted MTase of a type I R-M system. The DNA methylation sequence motif was confirmed by liquid chromatography coupled to tandem mass spectrometry (LC-MS/MS) analysis, and the methylation status was found to be dependent on growth conditions. Changes in the DNA methylation state could be correlated to changes in gene expression levels and pathogenicity in a *Galleria mellonella* infection model. Our results emphasize the role of DNA methylation as a regulator of gene expression in *P. aeruginosa* and illustrate how DNA methylation is involved in the regulation of phenotypic traits important for bacterial adaptation to conditions encountered in the infected host.

## RESULTS

### An HsdMSR type I restriction-modification system in *P. aeruginosa* PAO1.

*In silico* analysis of the *P. aeruginosa* PAO1 genome revealed the existence of a putative type I restriction-modification (R-M) system. This type I R-M system is composed of three subunits coded by three closely linked genes: PA2735 (*hsdM*), PA2734 (*hsdS*), and PA2732 (*hsdR*). Whereas the *hsdR* (PA2732) and putative MTase *hsdM* (PA2735) genes show 78% and 82% similarity, respectively, to their homologs found in *Klebsiella pneumoniae*, the *hsdS* gene (PA2734) shares 85% similarity with the gene encoding the restriction enzyme specificity protein found in *Xylella fastidiosa* (37, 38).

Bacteria use R-M systems as a defense against invasion by foreign DNA (10). However, there seem to be alternative roles of the bacterial R-M systems that are less clear, some of which are correlated with pathogenicity (16, 39, 40). In order to study the role of the HsdMSR type I R-M system in PAO1 in more detail, we generated a nonpolar *hsdMSR* deletion mutant. Deletion of this type I R-M system did not lead to significant changes in growth under standard laboratory conditions (see Fig. S1 in the supplemental material).

10.1128/mBio.02312-16.1 FIG S1  Growth of PAO1 and the Δ*hsdMSR* mutant in LB medium plotted in logarithmic scale. Growth was measured spectrophotometrically at 600 nm. Differences in growth between both strains were not significant. Data represent the mean from three independent experiments. Download FIG S1, TIF file, 0.1 MB. Copyright © 2017 Doberenz et al. 2017 Doberenz et al. This content is distributed under the terms of the Creative Commons Attribution 4.0 International license.

### SMRT sequencing reveals the MTase recognition motif of the HsdMSR R-M system in *P. aeruginosa* PAO1.

To functionally profile the HsdMSR R-M system in PAO1, we first identified the DNA methylation pattern throughout the PAO1 genome. We therefore performed SMRT sequencing on DNA extracted from stationary-phase lysogeny broth (LB) cultures. Our analysis revealed that only a small fraction of adenosine moieties are methylated in the PAO1 genome. With chromosomal DNA from PAO1 as a template, 5,605 nucleotides exhibited significant variations in polymerase kinetics, with an interpulse duration ratio (IPD) of >1.4, which is characteristic for DNA modifications. Of those modified nucleotides, 3974 were identified as N^6^-methyladenine (m6A) based on their distinct kinetic fingerprint.

**FIG 1:**
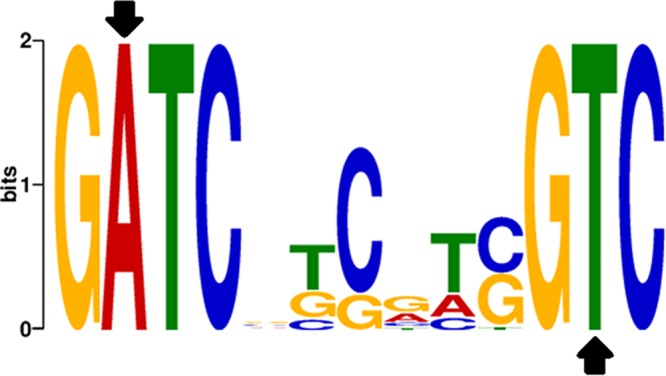
GATC(N)_6_GTC methylation motif found in *P. aeruginosa* PAO1 by the use of SMRT sequencing. Adenine methylation of this motif is strictly dependent on the presence of the DNA adenine MTase of the PAO1 HsdMSR system. The black arrows indicate the site of adenine methylation on both strands.

Next, we analyzed the local sequence context of the 3,974 m6A bases to determine if they were located within specific sequence motifs. Both by the use of the Pacific Biosciences motif finder as well as by the use of the MEME suite, we identified one G^m6^ATC(N)_6_GTC sequence motif (Fig. 1). (The underlined base indicates methylation on the opposite DNA strand.) At a detection quality cutoff value (QV) of ≥30, more than 80% of all m6A residues (3,181) could be assigned to this sequence motif. Out of these, 1,598 were found on the forward strand and 1,583 on the reverse strand of the PAO1 reference sequence (41). This nearly balanced strand-specific methylation pattern indicates that the GATC(N)_6_GTC sequence motif is for the most part fully methylated on both strands. Indeed, the GATC(N)_6_GTC sequence motif was found to be present 1,837 times in the PAO1 genome. A total of 1,463 of those sites (79.6%) were fully methylated under the chosen experimental conditions, 255 were hemimethylated, and 119 showed no detectable methylation. As depicted in Fig. S2 in the supplemental material, the DNA methylation motif was homogenously distributed across the genome, and there was no enrichment of motifs in the promoter regions of the PAO1 genome. In total, 541 promoters were fully or hemimethylated. Of note, also the hemimethylated and nonmethylated sites were homogeneously distributed across the genome. Interestingly, the frequencies of the presence of the GATC(N)_6_GTC sequence motif were comparable between PAO1 and PA14, indicating that this motif did not further evolve in PAO1 to serve particular functions. (PA14 does not possess a corresponding MTase.) However, the motif was less often found in the accessory genome of PAO1 (9.9% instead of the expected 12.1%), which to a large extent has been acquired by horizontal gene transfer.

10.1128/mBio.02312-16.2 FIG S2  Circos plot of the m6A sites detected in *P. aeruginosa* PAO1 by the use of SMRT sequencing. The detection quality values (QV) of the forward (outer track) and reverse (inner track) strands are projected onto the PAO1 core (blue) and accessory (orange) PAO1 genome (60). The hemimethylated sites are highlighted in magenta, and the nonmethylated sites are shown as a separate track colored in green. The sum of methylated sites per promoter for the 541 (hemi-) methylated promoters is shown as a new track colored in black. The innermost track shows the log_2_ fold change (outer track, positive expression; inner track, negative expression) of the 92 differentially expressed genes in the Δ*hsdMSR* mutant compared to its respective PAO1 wild-type strain. The type I R-M system and additional genes involved in iron metabolism are emphasized. Download FIG S2, TIF file, 1.8 MB. Copyright © 2017 Doberenz et al. 2017 Doberenz et al. This content is distributed under the terms of the Creative Commons Attribution 4.0 International license.

We also performed SMRT sequencing on the Δ*hsdMSR* deletion mutant. This analysis showed a loss of adenine methylation within all identified sequence motifs throughout the whole genome. Our results suggest that the HsdM DNA adenine MTase recognizes the GATC(N)_6_GTC sequence motif to specifically methylate the DNA. Since no further significant variations in polymerase kinetics with an interpulse duration (IPD) ratio of >1.4 could be detected on adenines in the Δ*hsdMSR* deletion mutant, only one major adenine-specific DNA MTase appears to be present in PAO1.

### Quantification of DNA adenine methylation using LC-MS/MS analysis.

We established an alternative experimental setup to quantify DNA methylation of genomic or plasmid-derived DNA. For this purpose, LC-MS/MS multiple-reaction monitoring (MRM) analysis was used. DNA from stationary-phase-grown *P. aeruginosa* PAO1 and the Δ*hsdMSR* deletion mutant was isolated, and 0.5 to 1 µg was enzymatically digested into their single nucleosides, followed by separation via LC and detection of the relevant mass transitions corresponding to each nucleoside by MRM MS/MS. The measured signal intensities of each deoxyribonucleoside were normalized to a standard calibration curve and an internal standard spiked into all samples. In accordance with the SMRT sequencing results, no methylation was detected in the Δ*hsdMSR* deletion mutant background, indicating that HsdM is the only MTase responsible for DNA adenine methylation in PAO1. We furthermore found that throughout the whole PAO1 genome, the DNA m6A content was rare and was only detected in 0.115% ± 0.003% of the adenines. Taking into account that the whole PAO1 genome consists of 2,095,084 adenines on both strands and harbors 1,837 GATC(N)_6_GTC sequence motifs, which can be methylated on both strands, the methylation level of all target sequences throughout the PAO1 genome can be approximated to range from 65 to 85%.

### Verification of putative MTase activity and specificity.

To further confirm that the observed methylation levels were due to the activity of the HsdM MTase, we expressed the MTase subunit gene *hdsM* together with the predicted specificity domain *hsdS* gene on an inducible vector construct in the Δ*hsdMSR* deletion mutant (see Fig. S3 in the supplemental material). As expected, we observed no methylation if *hsdMS* was not present. However, already basal *hsdMS* expression (due to the leaky expression of *hsdMS* under noninducing conditions) restored the methylation level to PAO1 wild-type levels.

10.1128/mBio.02312-16.3 FIG S3  Overexpression of *hsdMS* restores DNA methylation in the Δ*hsdMSR* mutant. Shown are genome-wide m6A levels in the Δ*hsdMSR* mutant complemented with *hsdMS* cloned into an arabinose-inducible pHERD20T vector. “+ Ara” indicates the induction with 0.2% arabinose in liquid culture for 8 h. The empty vector pHERD20T was used as a negative control. The bars show the mean ± standard deviation from three biological replicates. Significance was calculated with a Bonferroni adjusted one-way ANOVA. ****, *P* ≤ 0.0001. Download FIG S3, TIF file, 0.1 MB. Copyright © 2017 Doberenz et al. 2017 Doberenz et al. This content is distributed under the terms of the Creative Commons Attribution 4.0 International license.

**FIG 2:**
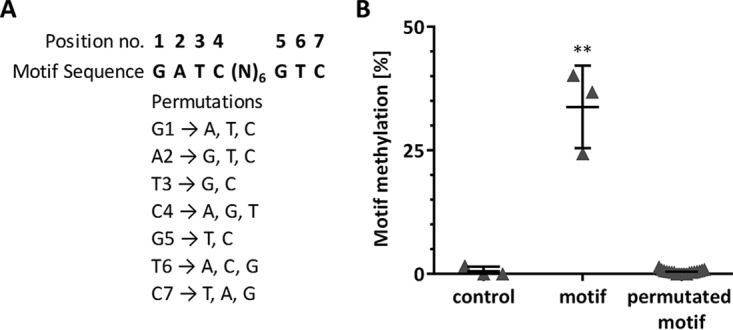
Analysis of motif conservation using LC-MS/MS analysis. (A) Single-nucleotide exchanges at the indicated positions of the cloned motif were subjected to LC-MS/MS analysis. (B) Targeted LC-MS/MS motif methylation analysis in PAO1 following the introduction of the permutated motif. The multiple-cloning site of the pUCP20 vector without the insertion of the DNA methylation sequence motif served as the negative control. The methylation status refers to *P. aeruginosa* PAO1 analyzed in the logarithmic growth phase (3 h). The mean and standard deviation are indicated by the horizontal lines and error bars, respectively. Data points for all 19 exchanged nucleotides are summarized in one graph. Experiments were done in triplicate. Significance was calculated by two-tailed unpaired Student’s *t* test. **, *P* ≤ 0.01.

In order to confirm the DNA methylation motif of the HsdM DNA m6A MTase, we cloned the 13 nucleotides of the DNA methylation motif into the multiple-cloning site of the pUCP20 vector and transformed PAO1 and the Δ*hsdMSR* deletion mutant with this construct, as well as with the empty vector control without the respective motif in the multiple-cloning site. EcoRI/HindIII treatment of the plasmids isolated from the respective strains revealed multiple-cloning site DNA fragments with and without the sequence motif, respectively (see Fig. S4 in the supplemental material). The resulting fragments were separated on a polyacrylamide gel and digested with DNA Degradase Plus. The nucleosides derived from the target sequences were purified and further analyzed by LC-MS/MS. As presented in Fig. 2B, the multiple-cloning site control DNA sequence that did not contain the motif showed no significant adenine methylation. However, when the motif was present, we observed a significant increase in the methylation level. In addition we tested the conservation of the motif. We found that the exchange of only one nucleotide within the conserved sequence region of the motif (Fig. 2A) was sufficient to abolish the methylation completely (Fig. 2B). This suggests very high sequence specificity for the corresponding MTase (PA2735) and its specificity domain protein, HsdS (PA2734).

10.1128/mBio.02312-16.4 FIG S4  Sequence background for determination of the relative methylation level using LC-MS/MS analysis. Sequence information of the multiple-cloning site (MCS) of the pUCP20 vector with and without the insertion of the GATC(N)_6_GTC motif. The 5′ and 3′ overhangs as a result of EcoRI and HindIII treatment are depicted in red. The motif sequence is shown in green. The sequence in blue is only present in the pUCP20 vector without the inserted motif. The total numbers of adenines present in the sequences of both strands are shown. fwd, forward (5′→3′); rev, reverse (3′→5′). Download FIG S4, TIF file, 0.1 MB. Copyright © 2017 Doberenz et al. 2017 Doberenz et al. This content is distributed under the terms of the Creative Commons Attribution 4.0 International license.

### The methylation pattern of sequence motifs implies nonfunctionality of the HsdMSR restriction enzyme.

The finding that only about two-thirds of the DNA methylation motifs were methylated throughout the PAO1 genome was unexpected, since R-M systems fulfill their role in the defense against invading foreign DNA by restricting those that exhibit respective nonmethylated DNA sequence motifs. This implies that the restriction enzyme HsdR might be nonfunctional in strain PAO1. Thus, to test for the functionality of the restriction enzyme subunit of the HsdMSR system, we introduced the *hsdRS* genes into the *hsdMSR* deletion mutant. Despite the presence of the HsdR protein (as determined by Western blotting [see Fig. S5 in the supplemental material]) in the deletion mutant, no change in growth was observed, indicating that the restriction enzyme subunit might be nonfunctional. Although it cannot be excluded that the modification domain is essential for restriction enzyme functionality, our results suggest that the HsdMSR system in PAO1 might have evolved to serve another function than to defend against invading foreign DNA.

10.1128/mBio.02312-16.5 FIG S5  Overexpression and detection of the C-terminal His-tagged putative restriction enzyme HsdR in PAO1 Δ*hsdMSR*. (A) The HsdR protein was cloned in combination with the HsdS protein domain into the inducible pME6032 vector. Shown is a representative SDS-PAGE analysis of extracted proteins from the PAO1 *hsdMSR* mutant with (lane 2) and without (lane 1) expression of the putative restriction endonuclease (REase) at an OD_600_ of 2. (B) Western blot analysis of the His-tagged REase with (lane 2) and without (lane 1) expression of the putative REase. Download FIG S5, TIF file, 0.2 MB. Copyright © 2017 Doberenz et al. 2017 Doberenz et al. This content is distributed under the terms of the Creative Commons Attribution 4.0 International license.

### Environmental conditions influence DNA methylation level.

A noncomplete methylation of DNA sequences might be a result of different synthesis levels of the MTase or of a modulation of the enzymatic activity under various environmental conditions. Loss of the restriction enzyme may also have permitted accumulation in the DNA methyltransferase gene by genetic drift. To explore this further, we cultivated wild-type PAO1 expressing the target DNA sequence motif in *trans* on the pUCP20 plasmid at different temperatures in LB medium and harvested the cells at different time points representing the logarithmic (3 h) or stationary (8 h) phase of growth. We indeed detected variations in the methylation level of the target sequence derived from the pUCP20 plasmid (see Fig. S6 in the supplemental material). Methylation was highest at 28°C in the stationary growth phase, with 53% ± 2.9% of the DNA sequence motifs being methylated, and lowest in the logarithmic phase at 42°C, with a methylation level of 16.7% ± 11.6%. In general we observed a decrease of the methylation level with increasing temperatures and an increased methylation level when bacteria reached the stationary phase.

10.1128/mBio.02312-16.6 FIG S6  DNA methylation is affected by environmental cues. Targeted DNA methylation analysis of the motif inserted into the multiple-cloning site of the pUCP20 plasmid in PAO1. The mean and standard deviation are indicated by the horizontal lines and error bars, respectively. Each data point represents one biological replicate. Significance is shown by asterisks and was calculated with a Bonferroni adjusted one-way ANOVA. **, *P* ≤ 0.01; ***, *P* ≤ 0.001. Download FIG S6, TIF file, 0.1 MB. Copyright © 2017 Doberenz et al. 2017 Doberenz et al. This content is distributed under the terms of the Creative Commons Attribution 4.0 International license.

### RNA sequencing reveals differentially regulated genes between PAO1 and the type I R-M system deletion mutant.

To explore whether DNA methylation via the HsdMSR system affects gene expression in PAO1, we performed a transcriptome analysis of the Δ*hsdMSR* deletion mutant and the wild-type strain PAO1. We cultivated the bacteria for 8 h until they reached early stationary growth phase, reflecting conditions under which DNA methylation is expected to be high. We found 92 differentially expressed genes in the PAO1 wild type compared to the Δ*hsdMSR* deletion mutant (see Table S1 in the supplemental material). Among them, 45 genes were upregulated and 47 genes downregulated. Out of the differentially regulated genes, 11 harbored a DNA methylation sequence motif in the promoter region. No difference in mRNA levels was found in the remaining 532 genes harboring a DNA methylation motif in their promoter region under the tested environmental conditions. Of note, 34 of the differentially expressed genes were involved in iron metabolism (Table S1).

10.1128/mBio.02312-16.8 TABLE S1  Differentially regulated genes ordered by PseudoCAP categories. Log_2_ fold changes (Log2FC) are shown for the PAO1 Δ*hsdMSR* mutant as well as the DNA methylation motif mutant (PAO1C5283613A) against the PAO1 wild type. Genes marked as boldface are involved in iron metabolism. Genes that are linked to iron metabolism were reported by D. Balasubramanian and K. Mathee (Hum Genomics 3:349-361, 2009, https://doi.org/10.1186/1479-7364-3-4-361) and U. Ochsner et al. (Mol Microbiol 45:1277-1287, 2002, https://doi.org/10.1046/j.1365-2958.2002.03084.x). n.d., no reads could be detected at least in one of the samples. Download TABLE S1, DOCX file, 0.04 MB. Copyright © 2017 Doberenz et al. 2017 Doberenz et al. This content is distributed under the terms of the Creative Commons Attribution 4.0 International license.

**FIG 3:**
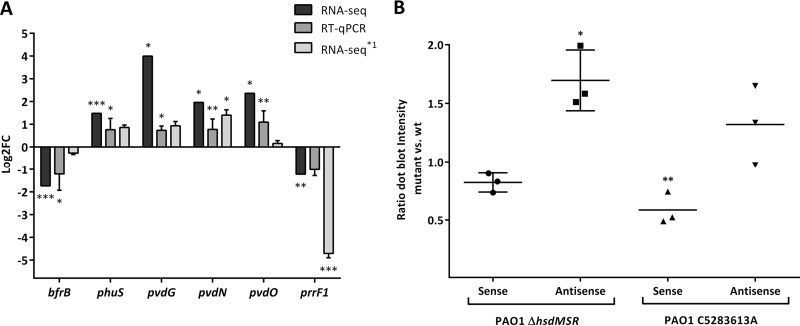
Verification of mRNA transcript levels using RT-qPCR and Northern dot blot analysis. (A) RT-qPCR analysis for representative genes that were identified as differentially expressed in the Δ*hsdMSR* mutant compared to the PAO1 wild-type (wt) strain by RNA-seq. The dark gray bars represent the mean log_2_ fold change values compared to a housekeeping gene (*rpsL*). The black and light gray bars show the log_2_ expression fold change levels observed by RNA-seq (Δ*hsdMSR* versus PAO1 wild type) and RNA-seq^*1^ (PAO1C5283613A versus PAO1 wild type). Statistical significance for the RNA-seq data was determined by negative binomial distribution analysis (DESeq package; R); significance levels are shown by asterisks: *, *P* ≤ 0.05; ***, *P* ≤ 0.001. The nonparametric Mann-Whitney *U* test was used to determine statistical significance of the RT-qPCR data: *, *P* ≤ 0.05; **, *P* ≤ 0.01. At least 4 independent biological replicates were analyzed. (B) Relative determination of the small regulatory RNA *prrF1* transcript ratio between the Δ*hsdMSR* mutant as well as the DNA methylation motif mutant and the corresponding PAO1 wild-type strain by Northern dot blot analysis. Each dot represents the corresponding ratio between the mutant and the wild type. Statistical significance between the antisense and sense ratios was determined using a Bonferroni corrected one-way analysis of variance (ANOVA): *, *P* ≤ 0.05; **, *P* ≤ 0.01.

In order to confirm the RNA sequencing results, we performed quantitative real-time PCR (RT-qPCR) experiments on a selection of genes that were found to be regulated. We included genes harboring at least one motif in the promoter region (*pvdN*, *pvdO*, *phuS*, and *prrF1*) and selected two genes (*bfrB* and *pvdG*) that do not have a motif in their promoter region but showed differential gene transcription. All of these genes are involved in iron metabolism. Whereas *bfrB* and *prrF1* were downregulated both in the transcriptome sequencing (RNA-seq) as well as in the RT-qPCR experiments, the other four genes were consistently upregulated in the Δ*hsdMSR* mutant compared to the PAO1 wild type (Fig. 3A).

### DNA methylation modulates expression of the small regulatory RNA *prrF1* in PAO1.

The finding that not all of the differentially expressed genes harbored a methylation motif in their promoter region indicates that secondary effects impact gene transcription in PAO1. Interestingly, the RNA sequencing data revealed downregulation of the small noncoding regulatory RNA *prrF1*, which harbors a methylation motif in the promoter region and is known to contribute to a global iron-sparing response via the repression of a set of gene targets (42). A more in-depth analysis of the RNA-seq data revealed that both strands of the *prrF1* coding gene were transcribed. The detected antisense transcript covered the entire sense TranScript. Of note, we found a strong and consistent inverse correlation between the sense gene and the corresponding antisense unit. This indicates that there might be a regulatory function of the antisense RNA. To explore this further, we expressed the *prrF1* gene in *trans* and performed strand-specific Northern blotting. Remarkably, whereas we found only a slight increase in sense *prrF1* gene expression in the wild type compared to the Δ*hsdMSR* mutant, there was increased antisense transcription of *prrF1* in the Δ*hsdMSR* mutant compared to the wild type (Fig. 3B). This suggests that missing DNA methylation of the GATC(N)_6_GTC sequence motif leads to an increased transcription of the antisense RNA and thus may decrease the sense target.

In order to further demonstrate that DNA methylation regulates *prrF1* gene expression in *P. aeruginosa* PAO1, we performed a site-directed mutagenesis and altered the DNA methylation motif in the promoter region of the small regulatory RNA. We then performed RNA-seq on the mutant strain (Table S1). As expected the *prrF1* gene was significantly downregulated in the promoter mutant. Furthermore, 9 of the 66 differentially expressed genes were involved in iron metabolism (*pvdA*, *pvdN*, *pvdS*, *bfd*, *fptA*, *fumC1*, *fagA*, *prrF1*, and *tonB1*) (Table S1), showing a significant enrichment of iron-related genes (one-sided Fisher’s exact test, *P* = 2.3e−05).

### The PAO1 Δ*hsdMSR* mutant exhibits a reduced-virulence phenotype and enhanced susceptibility against oxidative stress.

**FIG 4:**
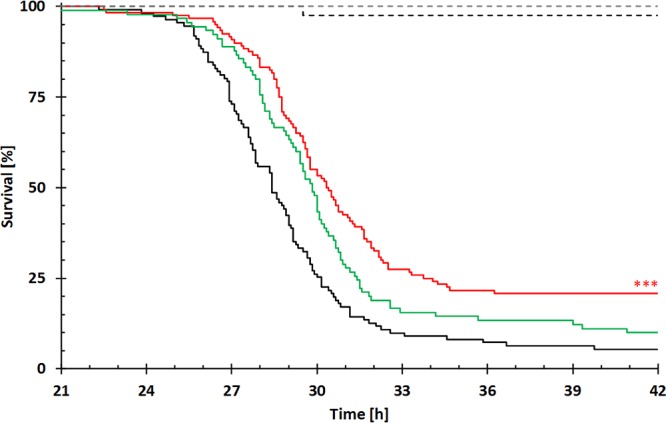
Relative survival rates of *P. aeruginosa*-infected *Galleria mellonella* larvae. Relative survival rates are depicted as Kaplan-Meier curves of the different groups with PAO1 with the empty pHERD20T vector control (black [*n* = 110]), PAO1 Δ*hsdMSR*(pHERD20T) (red [*n* = 120]), PAO1 Δ*hsdMSR*(pHERD20T::*hsdMS*) (green [*n* = 90]), PBS control (black dashed line [*n* = 40]), and untreated *G. mellonella* larvae (gray dashed line [*n* = 40]). The experiment was done in replicates on different days, and relative survival was plotted against the time of incubation. Significance was calculated by log-rank testing compared to the PAO1 wild-type group. ***, *P* ≤ 0.001.

To examine whether the loss of the type I R-M system affects the PAO1 virulence phenotype, we analyzed relative survival rates in a *Galleria mellonella* infection model. We found that the DNA methylation-deficient PAO1 mutant exhibited a marginally but significantly decreased virulence phenotype (50% lethal dose [LD_50_], 30.3 h) compared with the PAO1 wild-type (LD_50_, 28.4 h) (Fig. 4). When the DNA MTase gene was complemented in *trans* on the arabinose-inducible pHERD20T vector (i.e., its leaky expression resulted in an MTase activity that restored global DNA methylation to wild-type levels [Fig. S3]), the virulence phenotype of the PAO1 wild type could be partially restored.

We furthermore performed an oxidative stress assay (43) in which the bacteria were grown on LB soft agar plates and exposed to hydrogen peroxide spotted on sterile disks. The results are shown in Fig. S7 in the supplemental material. Indeed, we could detect a 14% increase of the inhibition zone in the Δ*hsdMSR* mutant background compared to the PAO1 wild type, demonstrating that the oxidative stress response is impaired in the PAO1 mutant that misses DNA methylation.

10.1128/mBio.02312-16.7 FIG S7  Oxidative stress response in PAO1 and the Δ*hsdMSR* mutant. The oxidative stress assay was performed with the PAO1 wild type and the Δ*hsdMSR* mutant. The mean and standard deviation of the hydrogen peroxide inhibition zone are indicated by the horizontal lines and error bars, respectively. The experiment was performed twice with three biological replicates. Each data point represents one biological replicate. The nonparametric Mann-Whitney *U* test was used to examine statistical significance. *, *P* ≤ 0.05. Download FIG S7, TIF file, 0.2 MB. Copyright © 2017 Doberenz et al. 2017 Doberenz et al. This content is distributed under the terms of the Creative Commons Attribution 4.0 International license.

## DISCUSSION

Third-generation SMRT sequencing technology can report MTase recognition motifs in bacterial genomes (34) and has been successfully used to search for DNA methylation patterns in bacteria like *Helicobacter pylori* and *Escherichia coli* (33, 44). In this study, we have used SMRT sequencing to uncover methylated adenines throughout the *P. aeruginosa* PAO1 genome, and methylation was found to be dependent on the activity of the *hsdM* DNA MTase encoded by PA2735. The SMRT sequencing data also gave detailed information on the site-specific methylation patterns. Methylated adenines were located within a highly conserved GATC(N)_6_GTC sequence motif that was to large extents methylated on both DNA strands. Nevertheless we also found hemimethylated and also nonmethylated sequence motifs. Current evidence indicates that undermethylation is caused by binding of proteins to the DNA, thereby protecting a site from methylation (21). Furthermore, local differences in the efficiency of the MTase might be due to an influence by the sequence context of the DNA methylation site, which may also contribute to undermethylation (6). There are several examples in *E. coli* in which a protein protects a site from Dam methylation, including the SeqA protection of methylation sites in the origin and OxyR protection of three GATC sites in the 5′ untranscribed region (UTR) of *agn43* (45).

Since HsdM MTase is part of a type I R-M system, it is surprising that the methylation state of these sites varied throughout the growth phases and did not exceed an overall relative methylation level of 65 to 85% under the experimental conditions applied in this study. For *Helicobacter pylori*, it was shown that a specific recognition sequence of a type I restriction-modification system was always fully methylated throughout the whole genome by applying SMRT sequencing (44). As lack of methylation makes the DNA vulnerable to cleavage by the corresponding restriction endonuclease, it is likely that the respective endonuclease is nonfunctional. Indeed, the sole inactivation of the MTase in PAO1 did not impair bacterial growth. However, we cannot exclude that motif recognition and thus the activity of the adjacent restriction endonuclease is strictly dependent on the simultaneous presence of the MTase. In the absence of a functional endonuclease, the modulation of methylation under changing environmental conditions could provide an effective mechanism for global and appropriate responses to particular environmental stimuli and may allow a fine-tuned epigenetic control.

The application of mass spectrometry in this study allowed a detailed determination of the relative methylation level of the adenines throughout the PAO1 genome and under various environmental conditions. Interestingly, we found enhanced activity of the MTase during the stationary growth phase and at low temperatures. We complemented our mass spectrometry and SMRT sequencing data with RNA-seq data on the *hsdMSR* mutant in order to gain more detailed information on whether DNA methylation in PAO1 might have a functional role on gene expression. Extensive studies of *E. coli*, *Vibrio cholerae*, and *Caulobacter crescentus* have significantly advanced our understanding of DNA methylation in bacteria (14, 15, 20); however, knowledge on the functional role of DNA methylation for the vast majority of prokaryotes is still very limited, and DNA methylation has rarely been acknowledged as a major regulator of gene expression (20). Here, functional profiling of the differentially regulated genes as a function of the presence of the HsdM MTase in the cell revealed an enrichment of genes involved in iron metabolism. Methylation of the amino group of adenine might affect gene transcription directly due to structural effects that impact the interaction of transcription factors with their target DNA (46). However, we found many differentially regulated genes that did not harbor a methylation motif in their promoter region and thus are likely to be affected indirectly. One interesting candidate for the mediation of these indirect effects is the small regulatory RNA *prrF1*, which is known to contribute to a global iron-sparing response via the repression of a set of gene targets (47). We found a methylation motif in the promoter region of *prrF1*, transcription of which was enhanced in the PAO1 wild type. Of note, RNA-seq revealed that there is an antisense transcript that spans the entire *prrF1* open reading frame. Interestingly, we found an enhanced transcription of the antisense RNA in the *hsdMSR* mutant as well as in a mutant harboring an altered nonmethylatable motif in the promoter region of *prrF1*. Antisense transcription thereby was inversely correlated with the sense transcript of the small regulatory RNA. Thus, the missing DNA methylation in the *prrF1* promoter region leads to facilitated transcription of the antisense *prrF1* transcript, which interferes with the activity of the sense transcript.

The roles of iron and *prrF1* in the regulation of (virulence) gene expression and oxidative stress response in *P. aeruginosa* have received considerable attention in the last few years (48). Of note, we observed slight but significant changes in oxidative stress resistance and decreased virulence in a *Galleria mellonella* infection assay for the Δ*hsdMSR* mutant as opposed to the PAO1 wild type. It is clear that low iron is used by a number of pathogens as a signal to sense the iron-sequestered environment of the human host and to turn on expression of not only iron acquisition systems and iron-sparing metabolic pathways but also bacterial virulence factors (49, 50). However, modulation of the low-iron response is very complex and involves not only the small regulatory RNA *prrF1* but also a second small regulatory RNA, *prrF2*, and also the global repressor Fur.

In the future, the broad application of SMRT sequencing and its combined use with RNA-seq will expand our understanding of DNA methylation in bacteria. Since the *P. aeruginosa* DNA MTases are not located within the core genome, this approach will also provide new insights into the scope and variety of DNA methylation in the species *P. aeruginosa*. Uncovering the functional roles of methylation will provide new insights into how strain-specific epigenetic changes may drive adaptation to particular environments.

## MATERIALS AND METHODS

### Bacterial strains, media, and reagents.

*P. aeruginosa* PAO1 (Lausanne) and* Escherichia coli* DH5α (51) cells were cultured in lysogeny broth (LB) medium at 37°C unless otherwise stated. When required, the following antibiotics were used: 100 µg/ml ampicillin, 400 µg/ml carbenicillin, and 60 µg/ml tetracycline.

### Construction of a PAO1 *hsdMSR* deletion mutant.

For inactivation of the *hsdMSR* locus in the *P. aeruginosa* PAO1 chromosome, an 881-bp fragment overlapping the first two codons of *hsdM* (PA2735) and a 725-bp fragment overlapping the last codon of *hsdR* (PA2732) were amplified by PCR using the primer pairs P1/P2 (CCGGGATCCTGGTAGCGCACTTGCA/GCGGTCGACTGCATTATCAGCGCTTCAA) and P3/P4 (CGGGTCGACATAGTCTGAAAACACTGTTGG/CCCAAGCTTAGCGAAGACCCACTCGG), respectively. These products were digested with BamHI-SalI and SalI-HindIII, respectively, and cloned within the BamHI-HindIII sites of the suicide vector pME3087 (52), giving plasmid pMEΔ*hsd*. The construct was verified by sequencing and then introduced into *P. aeruginosa* PAO1 by triparental mating, using the helper strain *E. coli* HB101(pRK2013). Merodiploids were resolved as previously described by Ye and colleagues (53). The resulting strain carried a Δ*hsdMSR* deletion fusing in the same reading frame the first two codons of *hsdM* with the last codon of *hsdR*, which was confirmed by PCR on genomic DNA.

The methylase activity disrupted in the Δ*hsdMSR* deletion mutant was restored by expressing the *hsdMS* genes in *trans*. To achieve this, the two genes were amplified by PCR with HotStar HiFidelity DNA polymerase (Qiagen, Hilden, Germany) from *P. aeruginosa* PAO1 genomic DNA using the primers CGAGCTCAGGAGGCTGATAATGCAGAAACGACAGC and AACTGCAGTCAGTCTTCAGCATCGGC (Eurofins, Ebersberg, Germany). SacI and PstI sites (underlined) were used to clone the PCR product into the arabinose-inducible expression vector pHERD20T (54), and the resulting pHERD20T::*hsdMS* construct was verified by sequencing before transformation into the *P. aeruginosa* PAO1 Δ*hsdMSR* mutant.

Endonuclease (HsdR) expression in the Δ*hsdMSR* mutant background was analyzed in *trans* by Western blotting. For this purpose, the *hsdRS* genes were cloned into the isopropyl-β-d-thiogalactopyranoside (IPTG)-inducible expression vector pME6032 by using the primer pair CGAGCTC**AGGAGGTGGCAG**GTGCTGCGTAATG (forward) and CGGGGTACCCTA***GTGGTGATGGTGATGATG***TCGCCCATGCGCAATCCT (reverse). The underlined primer sequences show sites for SacI and KpnI used for cloning, respectively. The boldface sequence of the forward primer indicates the ribosomal binding site, and the His tag introduced for Western blot analysis is emphasized as boldface italic sequence of the reverse primer. Following confirmation by sequencing, the construct was transformed into the PAO1 Δ*hsdMSR* mutant background. Western blot analysis was carried out according to Hwang and colleagues as described previously (55).

### Construction of a PAO1 methylation motif mutant.

The chromosomal region (5283517 to 5284445) flanking the *prrF* gene and the upstream methylation motif were cloned into a pUCP20 vector. The forward primer (GCTCTAGACTGGCGCACCTGAAGCAGC) consisted of an XbaI site, and the reverse primer (CGAGCTCTGAGGCCGACTACGCCTGG) contained a SacI site (underlined). Site-directed mutagenesis was then performed on the generated plasmid, pUCP20::*prrF*, by using the QuikChange II site-directed mutagenesis kit (Agilent Technologies, Frankfurt am Main, Germany) according to the manufacturer’s instructions. The primer pair GGCGACGATGCGGATAATGTGTTCATCATCA (forward) and TGATGATGAACACATTATCCGCATCGTCGCC (reverse) were used for the mutagenesis procedure. The result was a transversion of the first cytosine into an adenine of the inverse motif sequence GACGATGCGGATC. The construct was then used for a two-step marker-free mutagenesis protocol. Briefly, the insert was recloned into a pEX18Ap vector in an *E. coli* WM3064 background. The transformed *E. coli* donor strain was then used for homologous recombination with the *P. aeruginosa* PAO1 recipient strain. In the first step, clones were selected that incorporated the whole construct by testing for carbenicillin resistance (Carb^r^) and sucrose sensitivity (Suc^s^). For the second step, clones with the Carb^r^ Suc^s^ phenotype were grown for an additional 24 h and were selected for the opposite phenotype, indicating the occurrence of the second homologous recombination. Introduction of the mutation was furthermore verified by Sanger sequencing.

### Construction of a plasmid harboring the DNA methylation motif.

For the analysis of the methylation of the target DNA motif, a linker made of two complementary oligonucleotides (G**GATC**ATCGCT**GTC**T and CTAGA**GAC**AGCGAT**GATC**CTGCA [methylation motif underlined]) was cloned into the cloning vector pUCP20 (56). Briefly, 10 µM each primer was phosphorylated using T4 DNA ligase buffer (Roche) and T4 polynucleotide kinase (Thermo Scientific) according to the manufacturer’s instructions. The phosphorylated primers were pooled, denatured for 30 s at 95°C, and annealed with a temperature gradient starting with 95°C and gradually decreasing to 75°C in 2.5 min and finally from 75°C to 20°C in 28 min. The annealed linker was then cloned into pUCP20 treated with PstI and XbaI. After transformation into *E. coli* DH5α, the construct was isolated and sequenced prior its transformation into *P. aeruginosa* PAO1. Additional constructs harboring single-nucleotide exchanges in the motif region (in boldface in the primer sequence shown above) were generated (Fig. S4).

### SMRT sequencing and motif search.

SMRTbell template libraries were prepared according to the instructions from Pacific Biosciences, Menlo Park, CA, following the manufacturer’s procedure and checklist for 1-kb template preparation and sequencing, respectively. For preparation of 800-bp libraries, 4 µg of genomic DNA from overnight-grown cultures was sheared in microTubes using adaptive focused acoustics (Covaris, Woburn, MA). Size range was monitored on an Agilent 2100 Bioanalyzer from Agilent Technologies (Santa Clara, CA). DNAs were end repaired and ligated to hairpin adapters by applying components from the DNA/polymerase binding kit 2.0 from Pacific Biosciences, Menlo Park, CA. Reactions were carried out according to the manufacturer’s instructions. SMRTbell templates were exonuclease-treated for removal of incompletely formed reaction products. A mixture of exonuclease III and exonuclease VII (Affymetrix, High Wycombe, United Kingdom) was utilized. Conditions for annealing of sequencing primers and binding of polymerase to purified SMRTbell templates were assessed with the Calculator in RS Remote (Pacific Biosciences, Menlo Park, CA). SMRT sequencing was carried out on the PacBio RS (Pacific Biosciences, Menlo Park, CA). Altogether, for *P. aeruginosa* PAO1, three SMRTCells were obtained using C2 Chemistry in the mode of two 45-min movies, resulting in 239,307 reads with a mean read length of 2,408 bp.

Genome-wide detection of base modifications and the analysis of the local sequence context to determine their organization within specific sequence motifs were performed using the “RS_Modification_and_Motif_Analysis.1” protocol included in SMRTPortal version 2.3. Within this protocol, a FASTA export of GenBank entry AE004091.2 was used as the PAO1 genome reference. Standard parameters were applied, including Quiver as the consensus caller as well as a minimum modification QV of 30. Hereby, all recognized motifs show a larger modification score with a QV of 40.

### Transcriptomic analysis.

The RNA preparation procedure and comparative analysis of gene expression were carried out as previously described by Dötsch and colleagues (57). Total RNA was isolated from bacteria cultured for 8 h until they reached early stationary growth phase in LB rich medium at 42°C (optical density at 600 nm [OD_600_] of 4). (We assumed that methylation would be highest if bacteria are grown at elevated temperatures. However, this proved to be not the case [Fig. S6]. Nevertheless methylation of the motif was stable at 42°C under stationary-phase conditions.) RNA from 4 biological replicates of the PAO1 wild type, the PAO1 Δ*hsdMSR* mutant, and a PAO1 motif mutant (PAO1C5283613A) were pooled into one sample per strain and then subjected to the RNA extraction and preparation procedure for RNA sequencing. Statistical significance was determined as described previously by Khaledi and colleagues (63).

### RT-qPCR analysis.

Total RNA from *P. aeruginosa* PAO1 and the Δ*hsdMSR* mutant grown under the same conditions as for RNA-seq was isolated using the RNeasy Plus kit (Qiagen, Hilden, Germany). A residual quantity of genomic DNA was removed with the DNA-free DNase treatment and removal kit (Ambion, Austin, TX) according to the manufacturer’s instruction. The primer pairs for the tested genes were as follows (sequence from the 5′ to 3′ direction): *bfrB* (PA3531), forward, TCGCGATCAACCAGTACTTC, and reverse GCATGCTTCATCTCGTCGAT; *pvdG* (PA2425), forward, GCAGCACTTTTTCATCCGTG, and reverse, GACCTTGTCCTTGTGCCAG; *pvdN* (PA2394), forward, GTATCCACCAGCTCAACGC, and reverse, AGAAGGTGAAGCCCGAAGA; *pvdO* (PA2395), forward, CAGCGATGGCTACAACTTCA, and reverse, CGACCCACTCGTAGACGTT; *phuS* (PA4709), forward, TGATCCGGAATTCAACCTGC, and reverse, CCTCCCAGCTGGTTACGAT; *prrF1* (PA4704.1), forward, CTCAACTGGTCGCGAGAT, and reverse, CAAAGTGCCGGGTCAAAAA; and *rpsL* (PA4268), forward, TACGTCTGACCAACGGTTTC, and reverse, GTCCTTTACACGACCGCC.

Reverse transcription of the DNA-free total RNA into cDNA and further quantitative real-time PCR with the gene-specific primer pairs were performed with the QuantiFast SYBR green RT-PCR kit (Qiagen, Hilden, Germany) prepared in 96-well microtiter plates and analyzed with a Roche LightCycler 480 (Roche, Mannheim, Germany) according to the manufacturer’s instructions. Crossing point (CP) values were calculated using the second derivative maximum method of the LightCycler 480 software. Additional melting curve analysis was performed to check for primer dimerization. The calculated CP values were then used for quantification by the threshold cycle (ΔΔ*C*_*T*_) method described previously by Schmittgen and Livak (58). The specified target genes were normalized to the transcript level of the reference gene, *rpsL*, and the relative gene expression levels of the Δ*hsdMSR* mutant and PAO1 wild type were compared. The RT-qPCR experiment was performed in triplicate.

### Northern dot blot analysis.

To analyze strand-specific transcript levels of the small regulatory RNA *prrF1*, 5′-digoxigenin (DIG)-labeled RNA probes complementary to the sense or antisense transcript, respectively, were purchased from IBA (Göttingen, Germany). Probe 1 (GGCUGAUCUCGCGACCAGUUGAGUGACA) is complementary to the sense transcript, and probe 2 (UGUCACUCAACUGGUCGCGAGAUCAGCC) binds the antisense transcript. One to 10 µg DNA-free total RNA was diluted in 10 µl RNase-free water, denatured for 5 min at 95°C, cooled on ice, and spotted on an Amersham Hybond-N^+^ nylon membrane (GE Healthcare). The RNA was air dried and covalently bound on the membrane with UV light cross-linking at 254 nm. Northern hybridization of the DIG-labeled RNA probes was performed by the use of the DIG nucleic acid detection kit (Roche, Mannheim, Germany). Briefly the RNA sample cross-linked to the membrane was prehybridized with hybridization solution (5× SSC [1× SSC is 0.15 M NaCl plus 0.015 M sodium citrate], 10% [wt/vol] blocking reagent, 0.1% [wt/vol] *N*-laurylsarcosine, 0.02% [wt/vol] SDS) at 65°C for at least 3 h. Hybridization with 50 ng/ml of denatured 5′-DIG-labeled RNA probes was carried out overnight at 65°C in hybridization solution. The next day, the membrane was washed for 5 min at room temperature in high-salt hybridization washing solution (2× SSC, 0.1% [wt/vol] SDS) followed by an additional 15-min washing step at 65°C in low-salt hybridization washing solution (0.1% SSC, 0.1% [wt/vol] SDS). Finally, the membrane was rinsed in washing buffer (100 mM maleic acid, 150 mM NaCl, 0.3% [vol/vol] Tween 20 [pH 7.5]). Prior to immunodetection, the membrane was incubated for 30 min at room temperature in blocking solution (10% [wt/vol] blocking reagent, 100 mM maleic acid, 150 mM NaCl [pH 7.5]), and the anti-DIG antibody, diluted 1:5,000 in blocking solution, was added for 20 min. The membrane was washed twice for 15 min with washing buffer followed by 5 min of equilibration in detection buffer (100 mM Tris-HCl, 100 mM NaCl [pH 9.5]). Finally, the membrane was incubated with Amersham CDP-Star reagent (GE Healthcare, Buckinghamshire, United Kingdom) for 5 min before immunodetection using an Intas immunoblot imager system. Light signals were quantified using the ImageJ software version 1.48. Northern dot blot experiments were carried out on RNA isolated from bacteria grown under the same conditions as for the RNA-seq experiment in three independent biological replicates.

### LC conditions and MS parameters.

The LC-MS/MS measurements were performed on a Shimadzu Nexera high-performance liquid chromatography (HPLC) system (Shimadzu, Kyoto, Japan) coupled to a QTrap5500 triple quadrupole mass spectrometer equipped with an electrospray ionization (ESI) source (DuoSpray; ABSciex, Foster City, CA). DNA-derived deoxynucleosides were separated using a 50- by 4.6-mm Zorbax Eclipse XDB-C_18_ reverse-phase (RP) HPLC column (1.8-µm particle size) (Agilent, Santa Clara, CA). In addition, a 2-µm column saver (Supelco, Bellefonte, PA) and a C_18_ RP security guard (Phenomenex, Aschaffenburg, Germany) were combined upstream with the separation column. HPLC-grade water (J. T. Baker, Inc.) with 0.1% formic acid (J. T. Baker, Inc.) was used as LC solvent A. For solvent B, HPLC-grade methanol (J. T. Baker, Inc.) and 0.1% formic acid were used. The gradient started at 95% mobile phase A for 0.3 min, followed by a linear increase of solvent B up to 50% until 7.1 min. The solvent B concentration was held for another 1 min at 50% and decreased to 5% until 8.2 min. Equilibration with mobile phase A at 95% was performed until 11.2 min. The flow rate was set to 0.4 ml/min. The injection volume was set to 10 µl.

Samples were analyzed in the positive-ionization mode of the ESI source. Additional ESI parameters were as follows: source temperature, 400°C; ion spray voltage, 5.5 kV; curtain gas, 30 lb/in^2^; collision gas, 9 lb/in^2^; ion source gas 1, 60 lb/in^2^; and ion source gas 2, 75 lb/in^2^. Ion-specific parameters used in this study are presented in Table S2 in the supplemental material.

10.1128/mBio.02312-16.9 TABLE S2  Ion-specific parameters used for LC-MS/MS analysis. Shown are the parameters used for quantification of 2′-deoxyadenosine (dA), N^6^-methyl-2′-deoxyadenosine (m6dA), and tenofovir as the internal standard spiked into all samples prior to LC-MS/MS analysis. For the quantification, the quantifier fragment masses (Q) were used for quantification. Additional identifier masses (I) were used for the identification. DP, declustering potential; EP, exit potential; CE, collision energy; CXP, collision exit potential. Download TABLE S2, DOCX file, 0.03 MB. Copyright © 2017 Doberenz et al. 2017 Doberenz et al. This content is distributed under the terms of the Creative Commons Attribution 4.0 International license.

### Quantification of DNA-derived nucleosides.

Genomic or plasmid-derived DNA was isolated from planktonic cultures of *P. aeruginosa* PAO1 using the QIAprep plasmid minikit (Qiagen, Mannheim, Germany) as described by the manufacturer. Up to 1 µg of DNA was treated with 5 U DNA Degradase Plus (Zymo Research) per µg DNA according to the manufacturer’s instructions. An aliquot of the DNA was filled with HPLC-grade water up to a volume of 50 µl and mixed with 50 µl 100 ng/ml tenofovir (NIH, Bethesda, MD) as the internal standard. For the MS analysis, 10 µl of the sample was injected, corresponding to approximately 40 ng DNA. The intensities (peak area) of the DNA-derived nucleosides were measured and normalized to the internal standard. For quantification, the intensities were normalized with an external calibration curve starting with 34 pM with an increase to 5 µM in 2.5× steps for each nucleoside (obtained from Sigma-Aldrich, Seelze, Germany). For the quantification, quadratic regression and 1/× weighting were applied to calculate analyte concentrations in order to compare the ratio of N^6^-methyl-2′-deoxyadenosine (m6dA) to 2′-deoxyadenosine (dA) and compensate for various ion characteristics and matrix effects influencing the ionization efficiency. The threshold for the limit of detection (LOD) was set to a signal-to-noise (SN) ratio value of ≥3. The limit of quantification (LOQ) was considered a SN ratio value of ≥5 ± 20% accuracy of the corresponding calibration point. To determine the relative methylation content of the sample, the normalized intensity of m6dA was correlated to the sum of normalized intensity of m6dA and dA.

For analysis of the methylation level of a specific target sequence, the isolated plasmid harboring the sequence of interest with flanking sequence of the multiple-cloning site was treated with the restriction endonucleases EcoRI and HindIII and separated on a 10% polyacrylamide (PA) gel purchased from Bio-Rad (CA). For more information, see Fig. S4. The 51- to 58-bp fragments were cut out of the gel into 1-mm gel slices and dried with a SpeedVac (Eppendorf, Hamburg, Germany). The gel slices were soaked on ice for 15 min with 5 U DNA Degradase Plus in 25 µl buffer and overlaid with an additional 25 µl buffer. DNA degradation was carried out for 4 h at 37°C before enzyme inactivation at 70°C for 20 min. Nucleosides were purified by collecting the supernatant, desiccating the gel slices with 100 µl acetonitrile (Baker, Deventer, Netherland), and pooling the supernatant again, respectively. The supernatant of each sample was dried in a SpeedVac for 60 min and dissolved in 100 µl HPLC-grade water with 50 ng/ml tenofovir. Ten microliters of each sample was used for the MS analysis. The quantification of the nucleosides was done as described above and considering the sequence context of the analyzed fragment. Briefly, the relative methylation content was multiplied by the ratio of all adenines present in the sequence divided by the number of methylation sites for the MTase. The analysis was performed in triplicate.

### Oxidative stress assay.

According to the protocol previously described by Hassett and colleagues (59), which was modified as described by Nalca et al. (43), the sensitivity of PAO1 and the Δ*hsdMSR* mutant against oxidative stress was tested using an agar diffusion assay with hydrogen peroxide. For this purpose, a planktonic preculture was grown at 37°C overnight, and 100 µl of the culture with an OD_600_ of 6 was mixed with 3 ml of 0.6% (wt/vol) LB soft agar at 40°C. The suspension was poured on 1.5% (wt/vol) LB agar plates. Once the plates became solid, sterile filter paper disks were spotted on top of the agar, and 8 µl 30% (wt/vol) hydrogen peroxide solution (Roth, Karlsruhe, Germany) was pipetted on the disks. After further incubation at 37°C for 24 h, the zone of inhibition was determined. Two independent experiments with 3 biological and 3 technical replicates per strain were carried out. The mean of any technical replicates was summarized.

### Survival analysis of *P. aeruginosa*-infected *Galleria mellonella* larvae.

An overnight-grown planktonic preculture of *P. aeruginosa* was washed twice in sterile phosphate-buffered saline (PBS) and adjusted to a final concentration of 5 × 10^2^ CFU/ml. For each strain, 10 *Galleria mellonella* larvae were infected with 20 µl of the bacterial solution in the last left proleg. The infected larvae were further incubated at 28°C for 72 h. Survival was monitored with a webcam taking pictures every 5 min. The time of death was recorded when the respective larvae did not move after two frames. The experiment was done in replicates on different days, and relative survival was plotted for each sample against the time of incubation. Statistical analysis of the survival curves was calculated with log rank testing.
