# Supplementary figures and images for: Identification of a Pseudomonas aeruginosa PAO1 DNA Methyltransferase, Its Targets, and Physiological Roles
Source: mBio. 2017 Feb 21;8(1):e02312-16. doi: 10.1128/mBio.02312-16 (PMC5358918; doi:10.1128/mBio.02312-16)

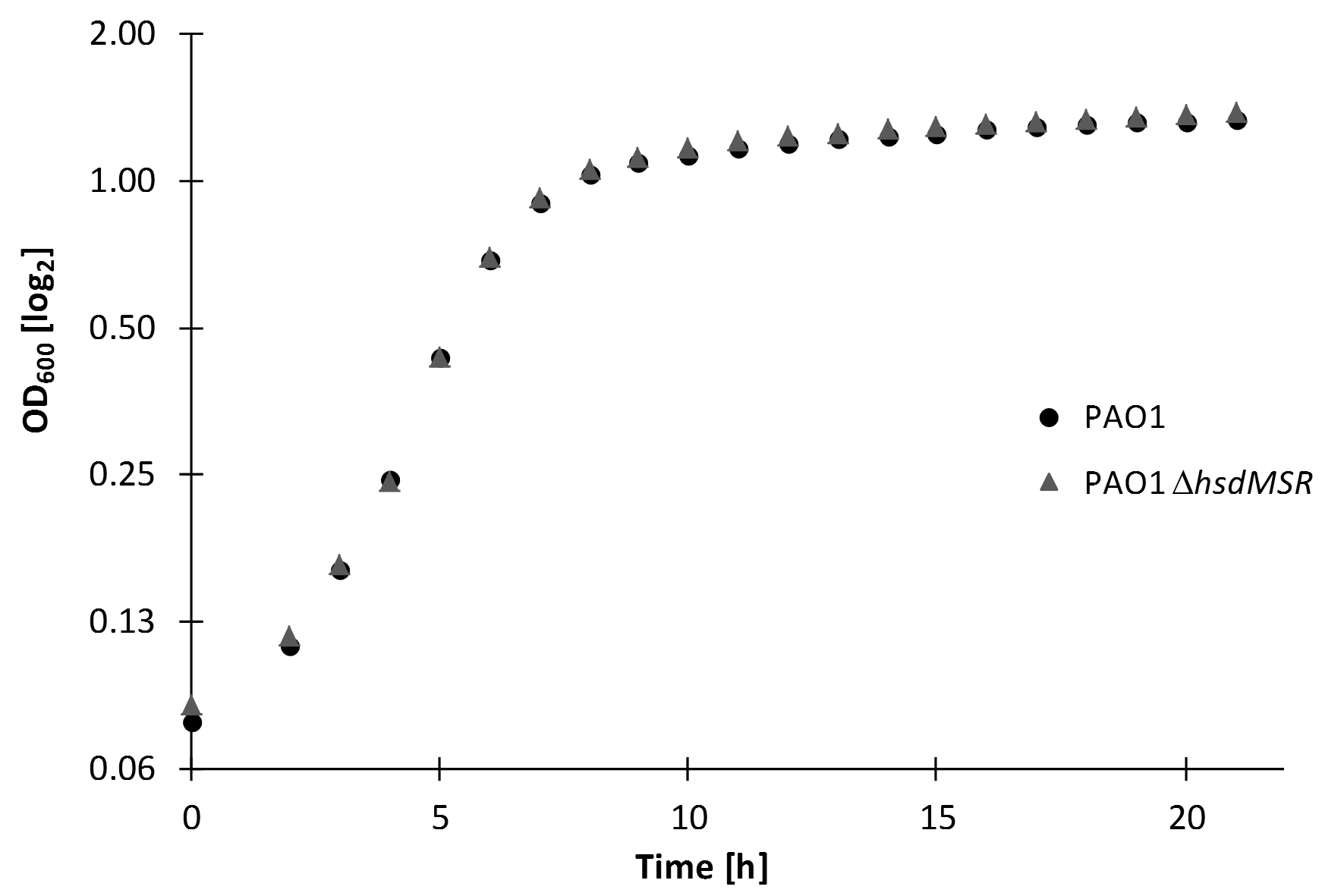

Supplement: FIG S1 [file mbo001173201sf1.tif]

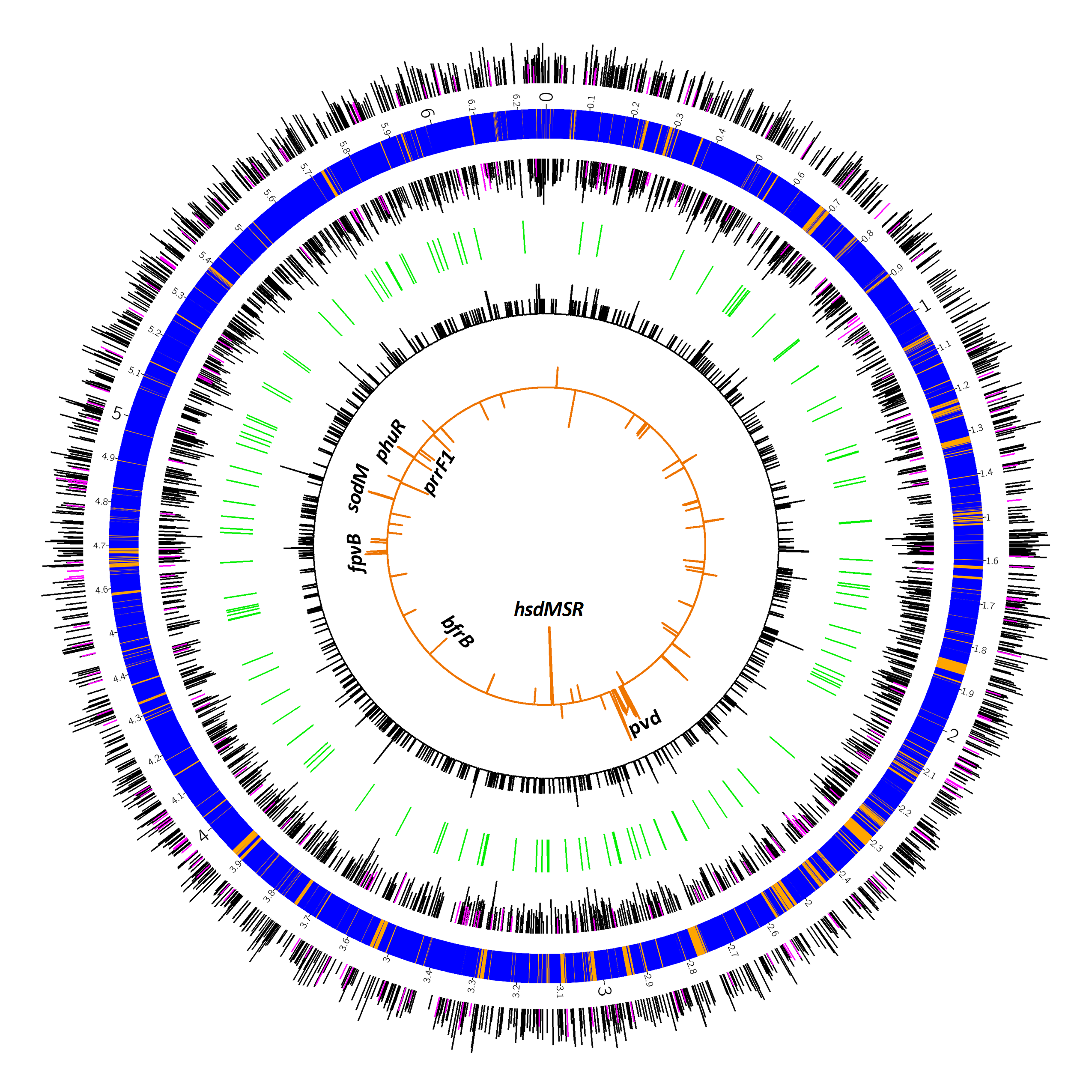

Supplement: FIG S2 [file mbo001173201sf2.tif]

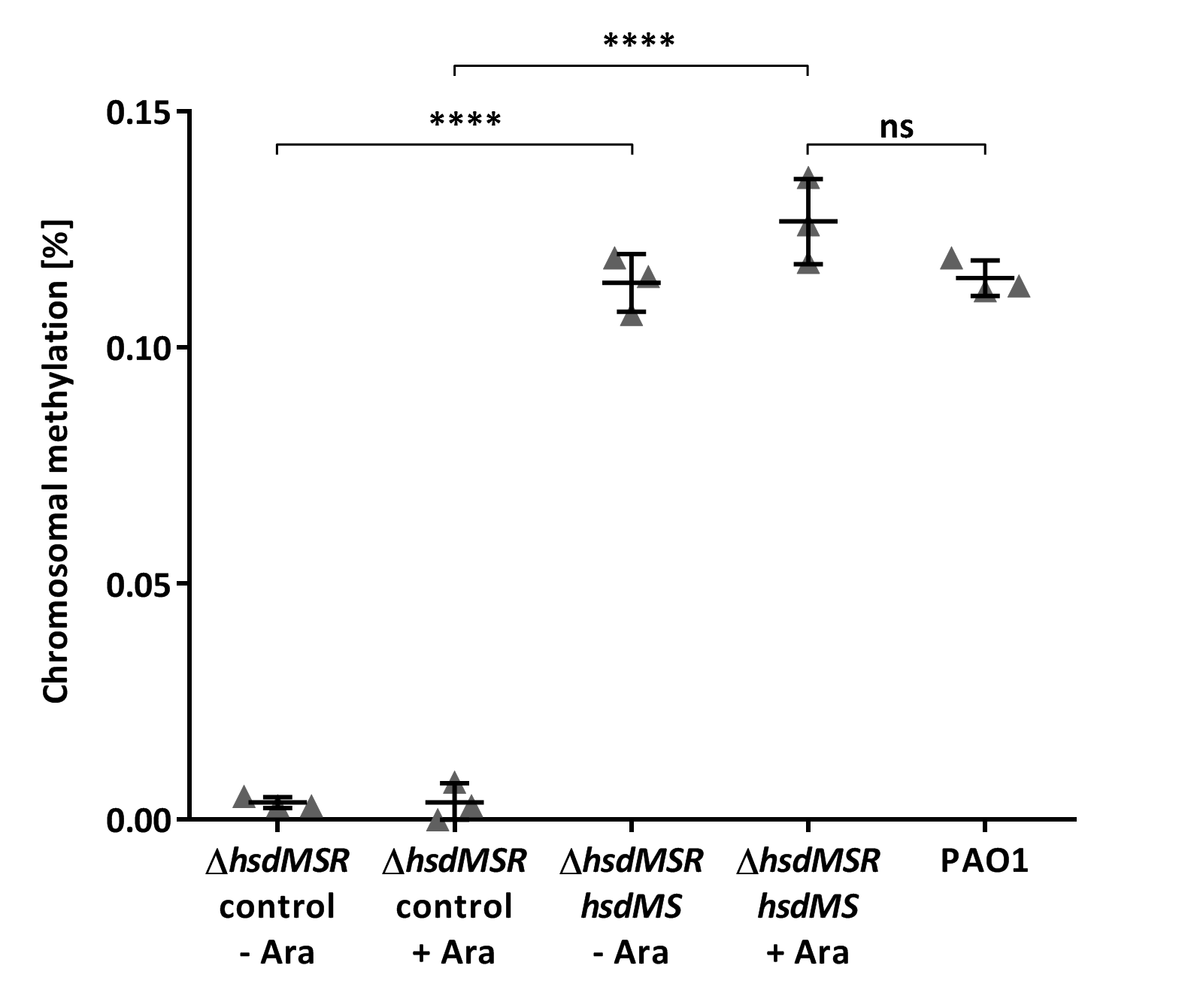

Supplement: FIG S3 [file mbo001173201sf3.tif]

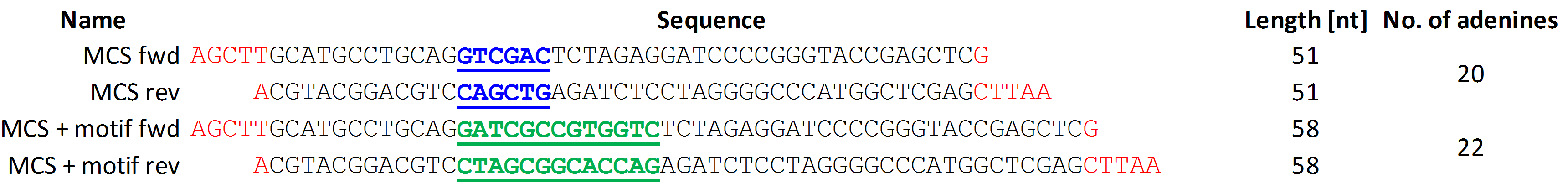

Supplement: FIG S4 [file mbo001173201sf4.tif]

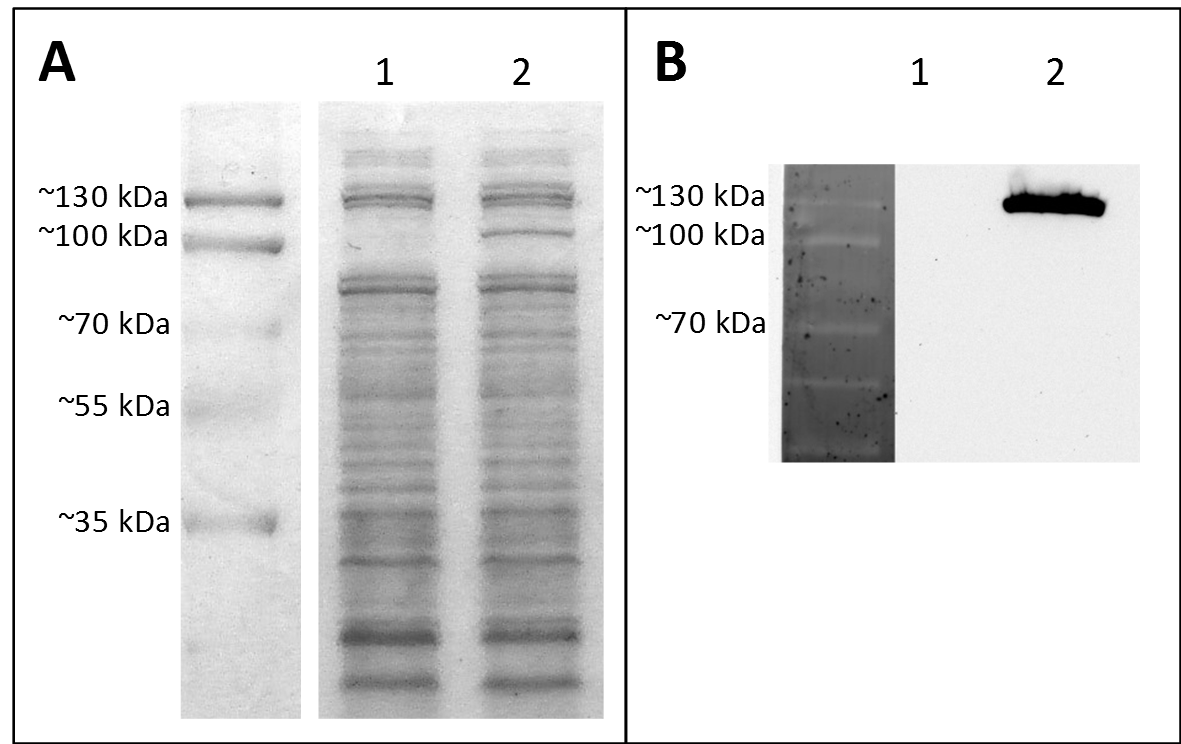

Supplement: FIG S5 [file mbo001173201sf5.tif]

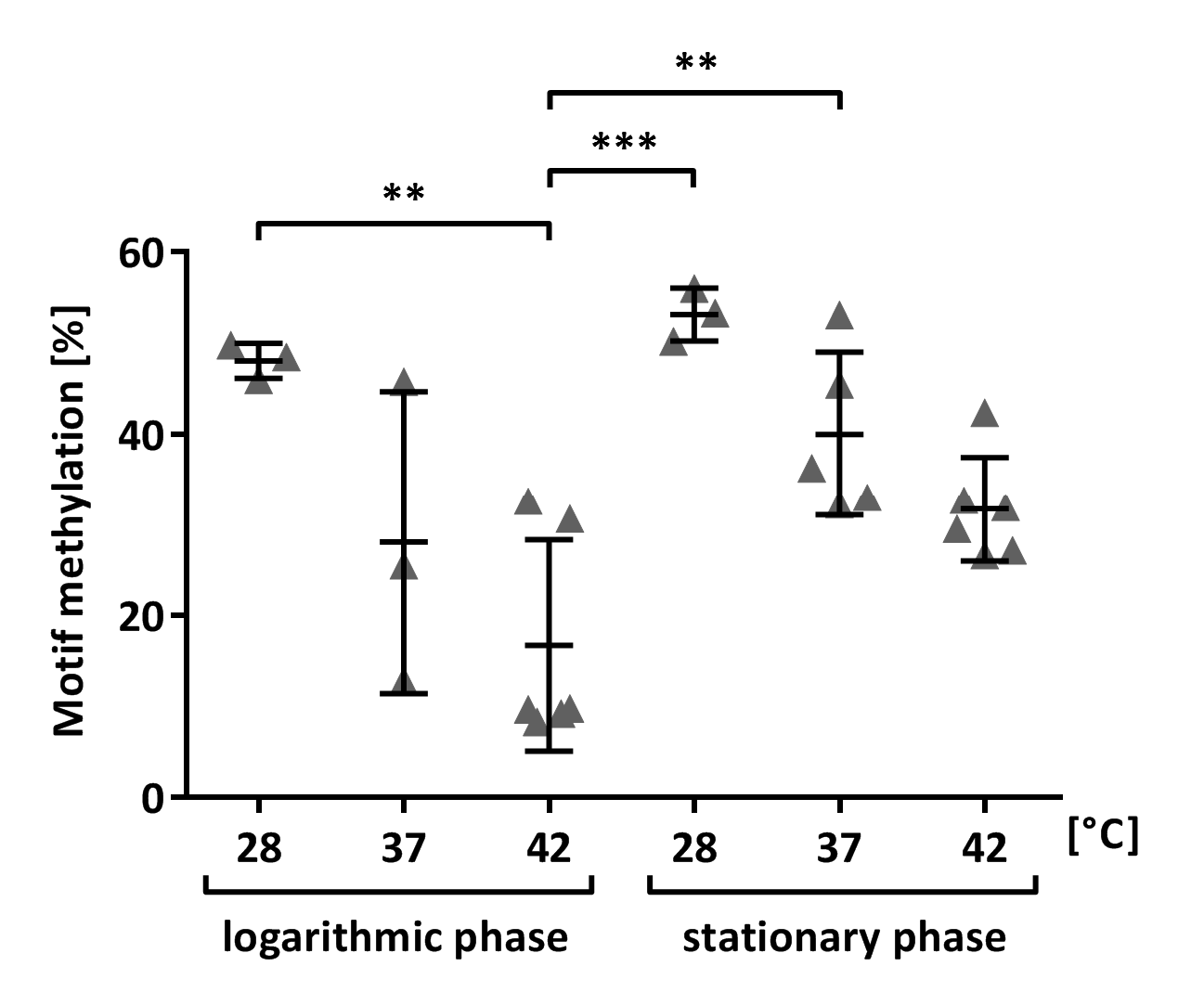

Supplement: FIG S6 [file mbo001173201sf6.tif]

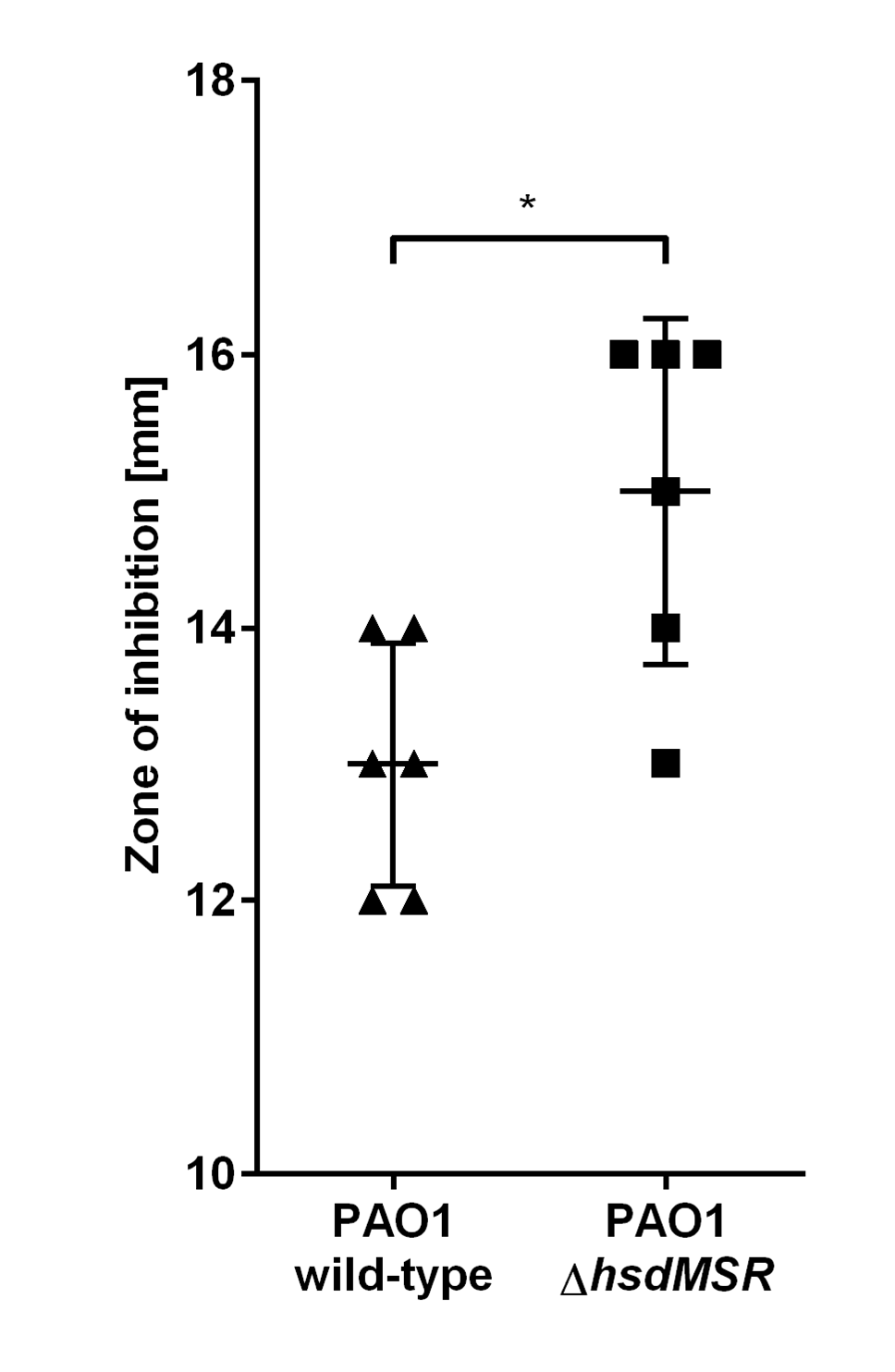

Supplement: FIG S7 [file mbo001173201sf7.tif]
