# Supplementary material for: Identification of a Pseudomonas aeruginosa PAO1 DNA Methyltransferase, Its Targets, and Physiological Roles
Source: mBio. 2017 Feb 21;8(1):e02312-16. doi: 10.1128/mBio.02312-16 (PMC5358918; doi:10.1128/mBio.02312-16)
Supplement: TABLE S1 [file mbo001173201st1.docx]

**Table S1: Differentially regulated genes ordered by PseudoCAP categories.** Log2 fold changes are shown for PAO1 Δ*hsdMSR* mutant as well as the DNA methylation motif mutant (PAO1C5283613A) against PAO1 wild-type. Genes marked as bold are involved in iron metabolism. Genes which are linked to iron metabolism were reported by (D. Balasubramanian and K. Mathee, Hum. Genomics 3:349-361, 2009, doi: 10.1186/1479-7364-3-4-361) and (U. Ochsner, P. Wilderman, A. Vasil, and M. Vasil, Mol. Microbiol. 45:1277-1287, 2002, doi: 10.1046/j.1365-2958.2002.03084.x). n.d., no reads could be detected at least in one of the samples.

| **PA no.** | **Gene name** | **Motif present in  promoter region** | **PseudoCAP category** | **Log2FC PAO1 Δ*hsdMSR*** | **Log2FC PAO1 C5283613A** |
| --- | --- | --- | --- | --- | --- |
| **PA2385** | ***pvdQ*** | no | Adaptation, Protection | 2.83 | 0.60 |
| **PA2386** | ***pvdA*** | no | Adaptation, Protection | 2.19 | 1.19 |
| **PA2392** | ***pvdP*** | no | Adaptation, Protection | 1.47 | 0.64 |
| **PA2394** | ***pvdN*** | yes | Adaptation, Protection | 1.96 | 1.40 |
| **PA2395** | ***pvdO*** | yes | Adaptation, Protection | 2.36 | 0.15 |
| **PA2400** | ***pvdJ*** | no | Adaptation, Protection | 1.70 | 0.79 |
| PA2411 |  | no | Adaptation, Protection | 1.47 | 0.01 |
| **PA2424** | ***pvdL*** | no | Adaptation, Protection | 2.34 | 0.71 |
| **PA2425** | ***pvdG*** | no | Adaptation, Protection | 4.00 | 0.93 |
| **PA4468** | ***sodM*** | no | Adaptation, Protection | 1.92 | 1.00 |
| PA1985 | *pqqA* | no | Biosynthesis of cofactors, prosthetic groups and carriers | -1.19 | -0.15 |
| PA2393 |  | no | Central intermediary metabolism | 2.55 | 1.54 |
| PA4563 | *rpsT* | no | Central intermediary metabolism | -2.24 | -0.35 |
| **PA4880** |  | no | Central intermediary metabolism | -1.75 | -0.64 |
| PA1172 | *napC* | no | Energy metabolism | -1.20 | -0.57 |
| PA1176 | *napF* | no | Energy metabolism | -1.09 | -0.37 |
| **PA4470** | ***fumC1*** | no | Energy metabolism | 1.93 | 1.61 |
| PA5304 | *dadA* | no | Energy metabolism | -1.05 | -0.23 |
| PA0049 |  | no | Hypothetical, unclassified, unknown | 1.54 | -0.31 |
| PA0161 |  | no | Hypothetical, unclassified, unknown | -2.83 | 0.76 |
| PA0613 |  | yes | Hypothetical, unclassified, unknown | -1.06 | -0.29 |
| PA0655 |  | no | Hypothetical, unclassified, unknown | -1.24 | 0.05 |
| PA1214 |  | no | Hypothetical, unclassified, unknown | -1.11 | 0.06 |
| PA1571 |  | no | Hypothetical, unclassified, unknown | -1.37 | -0.29 |
| PA1592 |  | yes | Hypothetical, unclassified, unknown | -1.24 | -0.10 |
| PA1597 |  | no | Hypothetical, unclassified, unknown | 1.06 | 0.25 |
| PA1968 |  | no | Hypothetical, unclassified, unknown | -1.48 | -0.10 |
| PA1969 |  | no | Hypothetical, unclassified, unknown | -1.36 | 0.13 |
| PA2033 |  | no | Hypothetical, unclassified, unknown | 1.37 | 0.29 |
| **PA2034** |  | no | Hypothetical, unclassified, unknown | 1.68 | 0.02 |
| PA2110 |  | no | Hypothetical, unclassified, unknown | 1.77 | 2.94 |
| PA2111 |  | no | Hypothetical, unclassified, unknown | 1.70 | -0.26 |
| PA2112 |  | no | Hypothetical, unclassified, unknown | 2.62 | -0.67 |
| PA2116 |  | no | Hypothetical, unclassified, unknown | 1.54 | 0.34 |
| PA2372 |  | no | Hypothetical, unclassified, unknown | -1.09 | -0.65 |
| **PA2384** |  | yes | Hypothetical, unclassified, unknown | 1.74 | 0.15 |
| **PA2412** |  | no | Hypothetical, unclassified, unknown | 1.76 | 0.87 |
| PA2575 |  | no | Hypothetical, unclassified, unknown | -1.23 | -0.30 |
| PA2730 |  | no | Hypothetical, unclassified, unknown | -2.68 | 0.02 |
| PA2731 |  | no | Hypothetical, unclassified, unknown | -2.07 | -0.23 |
| PA2732 |  | no | Hypothetical, unclassified, unknown | -5.83 | -0.02 |
| PA2733 |  | no | Hypothetical, unclassified, unknown | n.d. | -0.04 |
| PA2734 |  | no | Hypothetical, unclassified, unknown | n.d. | 0.05 |
| PA3786 |  | yes | Hypothetical, unclassified, unknown | -1.03 | -0.27 |
| **PA4220** | ***fptB*** | no | Hypothetical, unclassified, unknown | 1.21 | 0.63 |
| PA4377 |  | no | Hypothetical, unclassified, unknown | -1.46 | -0.51 |
| **PA4469** |  | no | Hypothetical, unclassified, unknown | 2.01 | 1.10 |
| **PA4471** | ***fagA*** | no | Hypothetical, unclassified, unknown | 1.28 | 2.00 |
| **PA4570** |  | no | Hypothetical, unclassified, unknown | 1.06 | 0.52 |
| PA4703 |  | no | Hypothetical, unclassified, unknown | -1.60 | -0.24 |
| **PA4897** | ***optI*** | no | Hypothetical, unclassified, unknown | 1.29 | 0.72 |
| **PA1301** |  | no | Membrane proteins | 1.52 | 0.78 |
| PA2114 |  | no | Membrane proteins | 2.01 | 0.25 |
| PA2404 |  | yes | Membrane proteins | 2.09 | 0.95 |
| PA2409 |  | no | Membrane proteins | 1.96 | 0.56 |
| **PA4467** |  | no | Membrane proteins | 1.36 | 0.72 |
| PA4765 | *omlA* | no | Membrane proteins | -1.32 | 0.18 |
| PA2407 |  | no | Motility & Attachment | 1.68 | 1.52 |
| PA0527.1 | *rsmY* | no | Non-coding RNA gene | -1.24 | -0.33 |
| PA0905.1 |  | no | Non-coding RNA gene | -1.50 | 0.20 |
| PA1324.1 |  | no | Non-coding RNA gene | -1.34 | -0.91 |
| PA1530.1 | *ffs* | no | Non-coding RNA gene | -1.58 | -0.86 |
| PA1804.1 |  | yes | Non-coding RNA gene | -1.15 | 0.23 |
| PA2736.1 |  | no | Non-coding RNA gene | -1.48 | -2.10 |
| PA2852.1 |  | no | Non-coding RNA gene | -1.20 | -0.71 |
| PA3133.2 |  | no | Non-coding RNA gene | -1.56 | -1.03 |
| PA3133.4 |  | no | Non-coding RNA gene | -1.36 | -0.83 |
| PA4277.2 |  | no | Non-coding RNA gene | -1.02 | -0.68 |
| **PA4704.1** | ***prrF1*** | yes | Non-coding RNA gene | -1.20 | -4.71 |
| PA4746.1 |  | no | Non-coding RNA gene | -1.11 | -0.78 |
| PA4937.1 |  | no | Non-coding RNA gene | -1.47 | -1.06 |
| PA4937.2 |  | no | Non-coding RNA gene | -1.04 | -1.09 |
| PA5181.1 |  | no | Non-coding RNA gene | -1.46 | 0.44 |
| **PA2402** |  | yes | Putative enzymes | 1.96 | 0.73 |
| PA2735 |  | no | Putative enzymes | n.d. | 0.29 |
| PA4022 | *exaC2* | no | Putative enzymes | -1.24 | 1.03 |
| **PA4709** | ***phuS*** | yes | Putative enzymes | 1.48 | 0.86 |
| PA0627 |  | no | Related to phage, transposon, or plasmid | -1.16 | -0.62 |
| **PA2399** | ***pvdD*** | no | Secreted Factors (toxins, enzymes, alginate) | 1.60 | 0.55 |
| PA0961 |  | no | Transcriptional regulators | -1.62 | 0.36 |
| **PA1300** |  | no | Transcriptional regulators | 1.35 | 0.92 |
| PA2468 | *foxI* | no | Transcriptional regulators | 1.11 | 0.34 |
| **PA2686** | ***pfeR*** | no | Transcriptional regulators | 1.05 | 0.94 |
| **PA4227** | ***pchR*** | no | Transcriptional regulators | 1.19 | 0.72 |
| PA4315 | *mvaT* | no | Transcriptional regulators | -1.01 | -0.38 |
| PA2619 | *infA* | no | Translation, post-translational modification, degradation | -1.03 | -0.10 |
| **PA0931** | ***pirA*** | no | Transport of small molecules | 1.25 | 0.41 |
| PA2113 | *opdO* | no | Transport of small molecules | 2.67 | 0.11 |
| **PA3531** | ***bfrB*** | no | Transport of small molecules | -1.74 | -0.27 |
| PA4156 |  | no | Transport of small molecules | 1.67 | 1.03 |
| **PA4168** | ***fpvB*** | no | Transport of small molecules | 1.51 | 0.41 |
| **PA4710** | ***phuR*** | no | Transport of small molecules | 1.42 | 0.69 |
