# Supplementary material for: Identification of a Pseudomonas aeruginosa PAO1 DNA Methyltransferase, Its Targets, and Physiological Roles
Source: mBio. 2017 Feb 21;8(1):e02312-16. doi: 10.1128/mBio.02312-16 (PMC5358918; doi:10.1128/mBio.02312-16)
Supplement: TABLE S2 [file mbo001173201st2.docx]

Table S2: Ion specific parameters used for LC-MS/MS analysis. Parameters used for quantification of 2’-deoxyadenosine (dA), N6-methyl-2’-deoxyadenosine (m6dA) and Tenofovir as the internal standard spiked into all samples prior LC-MS/MS analysis. For the quantification the quantifier fragment masses (Q) were used for quantification. Additional identifier masses (I) were used for the identification. DP, declustering potential; EP, exit potential; CE, collision energy; CXP, collision exit potential.

| Analyte | Precursor [m/z] | Fragment mass [m/z] | | Dwell time [ms] | DP [V] | EP [V] | CE [V] | CXP [V] |
| --- | --- | --- | --- | --- | --- | --- | --- | --- |
| dA | 252.043 | 119.00 | (I) | 50 | 56 | 10 | 59 | 12 |
|  |  | 135.99 | (Q) | 50 | 156 | 10 | 21 | 12 |
| m6dA | 266.062 | 123.00 | (I) | 50 | 71 | 10 | 55 | 10 |
|  |  | 107.97 | (I) | 50 | 71 | 10 | 75 | 12 |
|  |  | 150.02 | (Q) | 50 | 71 | 10 | 23 | 12 |
| Tenofovir | 287.984 | 176.00 | (Q) | 50 | 16 | 10 | 35 | 14 |
|  |  | 270.00 | (I) | 50 | 16 | 10 | 27 | 18 |
